# Severe Bleeding From Portal Hypertension in a Young Patient With Common Variable Immunodeficiency-Associated Nodular Regenerative Hyperplasia

**DOI:** 10.14309/crj.0000000000001891

**Published:** 2025-11-17

**Authors:** Vijayvardhan Kamalumpundi, Darine Daher, Zongming Eric Chen, Sarah Khan, Navreet Chowla

**Affiliations:** 1 Department of Internal Medicine, Mayo Clinic, Rochester, MN; 2 Department of Laboratory Medicine and Pathology, Mayo Clinic, Rochester, MN; 3 Division of Gastroenterology and Hepatology, Mayo Clinic, Rochester, MN

**Keywords:** common variable immunodeficiency, nodular regenerative hyperplasia, noncirrhotic portal hypertension, peristomal bleeding, immunodeficiency

## Abstract

Common variable immunodeficiency (CVID) is a primary immunodeficiency disorder causing hypogammaglobulinemia. Although rare, nodular regenerative hyperplasia is the most common liver complication in CVID, which can progress to noncirrhotic portal hypertension. We report a case of a young male with CVID presenting with recurrent parastomal bleeding secondary to noncirrhotic portal hypertension from CVID-associated nodular regenerative hyperplasia.

## INTRODUCTION

Common variable immunodeficiency (CVID) is a group of genetic disorders characterized by defective B-cell differentiation and hypogammaglobulinemia, resulting in recurrent infections and multiorgan involvement including the liver. Hepatic complications occur in at least 10% of patients with CVID, with nodular regenerative hyperplasia (NRH) being the most common subtype. We present a case of a young patient with chronic stomal variceal bleeding secondary to CVID-associated NRH.^1,2^

## CASE REPORT

A 24-year-old man with a history of CVID on monthly subcutaneous immunoglobulin, and a confirmed STAT1 gain-of-function mutation by whole genome sequencing (c.1154C>T (p.Thr385Met), presented with parastomal bleeding. History is notable for CVID enteropathy, inflammatory rectal pseudopolyposis status post abdominoperineal resection with permanent colostomy one year prior, and noncirrhotic portal hypertension (NCPH) secondary to NRH.

Physical examination revealed no signs of encephalopathy or ascites. Bleeding originated from congested mucosa at the stomal site, appearing deep red, engorged, and oozing. Laboratory results showed hemoglobin 9.1 g/dL (baseline 10.2), blood urea nitrogen 14 mg/dL, serum creatinine 0.97 mg/dL, aspartate aminotransferase 65 U/L, alanine aminotransferase 36 U/L, alkaline phosphatase 310 U/L, total bilirubin 0.5 mg/dL, direct bilirubin, <0.2 mg/dL, and international normalized ratio 1.1. Abdominal and pelvic computed tomography (CT) imaging demonstrated increased portal circulation decompressing through the inferior mesenteric vein into parastomal varices, with interval variceal enlargement compared with one year prior.

The patient had undergone percutaneous sclerotherapy 8 months earlier for a similar bleeding episode. During the current admission, he underwent Sotradecol sclerotherapy and coil embolization of the stomal varices again but continued to experience oozing into the stomal bag, requiring 4 units of packed red blood cells. Repeat CT angiography revealed no arterial source of gastrointestinal bleeding to explain additional blood loss.

**Figure 1.**
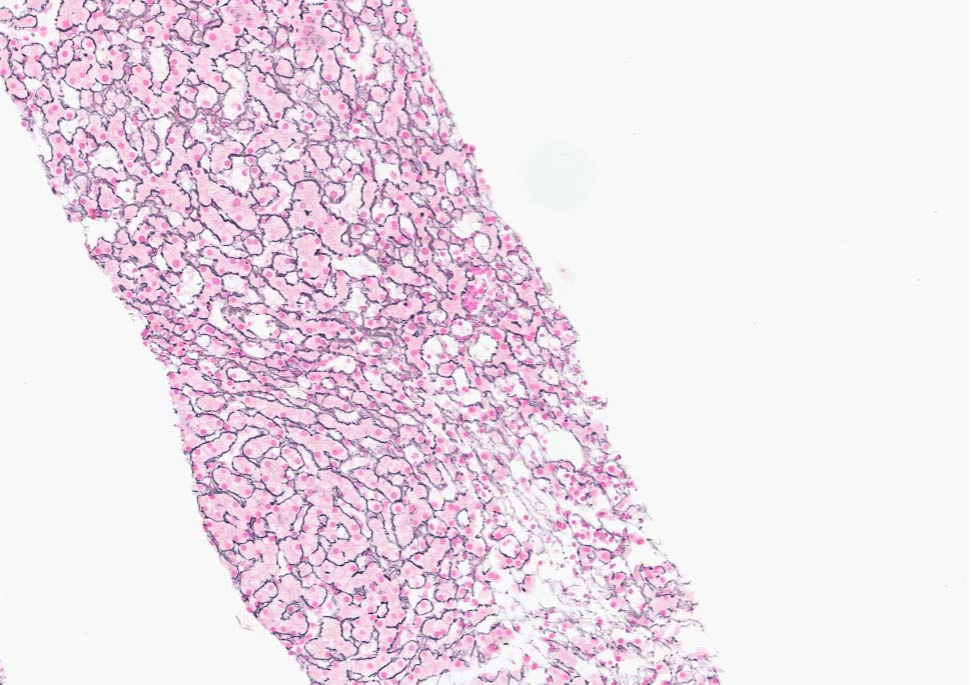
Liver biopsy shows nodular regenerative hyperplasia. In this reticulin stain, 2 nodules composed of 2-cell-thick hepatocyte plates are bordered by atrophic hepatocytes. Original magnification ×20.

**Figure 2.**
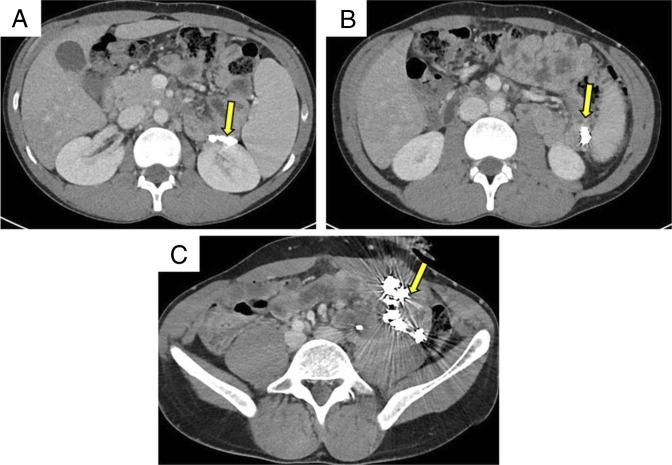
(A–C) Transverse sections of abdominal computed tomography showing embolization coils adjacent to (A) left kidney, (B) left retroperitoneum, and (C) left lower quadrant in the L. paracolic gutter (yellow arrow).

He was started on a continuous octreotide infusion. Colonoscopy revealed normal colonic mucosa with no evidence of bleeding in the terminal ileum or colon. Esophagogastroduodenoscopy and triple-phase CT enterography demonstrated very small esophageal varices and enlarged parastomal varices, without definite vascular malformation or enhancing mass within the small bowel. The patient did not require intubation for the procedure. Anemia was therefore attributed to continuous, slow parastomal variceal bleeding. Consequently, the patient underwent transhepatic coil embolization of stomal varices, resulting in successful resolution of the bleeding (Figure 1). Transjugular liver biopsy with pressure measurements revealed a hepatic venous pressure gradient of 12–13 mm Hg (normal range: 1–5 mm Hg), indicating portal hypertension. Biopsy demonstrated congestive hepatopathy, focal/nonspecific portal and lobular inflammation, and patchy sinusoidal fibrosis, consistent with NRH (Figure 2).

**Figure 3.**
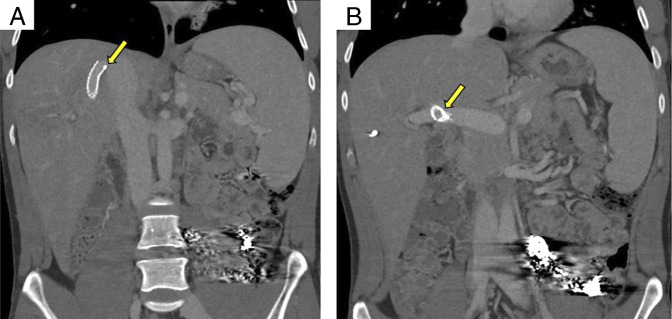
(A and B) Coronal section of abdominal computed tomography showing transjugular intrahepatic portosystemic shunt stent between right portal vein and right hepatic vein.

Initially, given the patient's young age and the risk of future occlusion and infection, a transjugular portosystemic shunt (TIPS) and surgical shunt was deferred. However, 1 month later, the patient re-presented with recurrent stomal bleeding, and TIPS was pursued given the refractory nature of the parastomal varices (Figure 3). The portosystemic gradient reduced from 10 to 4 mm Hg after TIPS. His recovery was complicated by profound immune thrombocytopenia to 3 ×10^9^/L and blood loss anemia as the procedure required multiple passes. He was discharged with strict return precautions. Follow-up was arranged with allergy/immunology to evaluate the use of ruxolitinib and/or bone marrow transplantation for further CVID management. Platelet counts have since recovered to baseline, and the patient remains stable from a bleeding standpoint 6 month later.

## DISCUSSION

This is the first reported case of a young patient with severe and recurrent stomal bleeding from congested mucosa and varices secondary to NCPH from CVID-associated NRH treated with TIPS.

NRH is primarily a histopathologic diagnosis, characterized by formation of small regenerative hepatic nodules without significant fibrosis. Nodules are composed of hyperplastic hepatocytes that can cause compression of the sinusoids.^3^ The pathogenesis of NRH in CVID remains unclear but is hypothesized to involve sinusoidal lymphocytic infiltration, particularly by cytotoxic T cells, leading to endothelial injury, aberrant nodule formation, and progression to NCPH or even cirrhosis.^4^ Other potential mechanisms may include autoimmunity, CVID-enteropathy, and dysregulation of the gut-liver axis.^5^

There are no established treatments to slow progression of NRH in CVID. Azzu et al showed that hepatic involvement portends a higher mortality rate (28% vs 6% respectively), compared with those without hepatic involvement.^2^ Liver transplantation has been performed for CVID-associated end-stage liver disease, though was associated with poor outcomes due to disease recurrence. Only a few cases have reported the effect of CVID on the liver. A brief review highlighted that rituximab had favorable effects on alkaline phosphatase, led to resolution of ascites and a partial or complete response in CVID-related granulomatous liver disease.^6–9^ However, rituximab was used for interstitial lung disease in these studies and not for a primary liver indication. Oral budesonide was shown in a case of CVID-associated NRH to improve liver chemistries, though it was unclear if this prevented disease progression.^10^

Bleeding from NCPH due to CVID-associated NRH carries high mortality. A retrospective study found that 41% of CVID patients with NCPH died from bleeding complications, though it is unclear if any underwent TIPS.^11^ While TIPS has been used in refractory cases, mortality remains significant, with a multicenter study reporting 46% mortality in 13 patients with CVID due to septic shock within 4 years of TIPS.^12–14^ Given the refractory nature of our patient's parastomal bleeding, TIPS was pursued despite risks such as shunt dysfunction and infection. Notably, NCPH in CVID is associated with higher rates of autoimmune cytopenia, enteropathy, and lymphadenopathy compared with patients with CVID without NCPH.^11^ Our patient's history of CVID-enteropathy and immune-related thrombocytopenia suggests a severe, multisystem manifestation of CVID.

Last, only 5% of CVID cases have an identifiable genetic variant. This patient carries a STAT1 gain-of-function mutation, which increases the risk of chronic mucocutaneous candidiasis. Ruxolitinib (a JAK inhibitor) has demonstrated remarkable improvements in enteritis, natural killer cell function, and dysregulated T-cell polarization in patients with STAT1 mutations.^15^ Given the potential involvement of the gut-liver axis in the pathogenesis of CVID-associated NRH, ruxolitinib could be a promising treatment of NRH, although no studies have yet explored this hypothesis.

In summary, this case illustrates a young patient with severe parastomal variceal bleeding due to NCPH from CVID-associated NRH. We hope this case contributes to the literature on CVID-associated liver complications and offers insight into potential treatment approaches.

## DISCLOSURES

Author contributions: Conceptualization: S Khan; Writing—original draft preparation: V Kamalumpundi, D Daher; Writing—review and editing: All authors; Figure Creation: V Kamalumpundi, ZE Chen; Supervision: S Khan, N Chowla, V Kamalumpundi is the article guarantor.

Financial disclosure: None to report. The authors declare no conflict of interest.

Informed consent was obtained for this case report.

## ABBREVIATIONS:

CT: computed tomography
CVID: common variable immunodeficiency
NCPH: noncirrhotic portal hypertension
NRH: nodular regenerative hyperplasia
STAT1: signal transducer and activator of transcription 1
TIPS: transjugular intrahepatic portosystemic shunt
